# Genes divided according to the relative position of the longest intron show increased representation in different KEGG pathways

**DOI:** 10.1186/s12864-024-10558-x

**Published:** 2024-06-28

**Authors:** Pavel Dvorak, Viktor Hlavac, Vojtech Hanicinec, Bhavana Hemantha Rao, Pavel Soucek

**Affiliations:** 1 Department of Biology, Faculty of Medicine in Pilsen, Charles University, Alej Svobody 76, 32300 Pilsen, Czech Republic; 2 Biomedical Center, Faculty of Medicine in Pilsen, Charles University, Alej Svobody 76, 32300 Pilsen, Czech Republic; 3 Institute of Medical Genetics, University Hospital Pilsen, Dr. Edvarda Benese 13, 30599 Pilsen, Czech Republic; 4 Toxicogenomics Unit, National Institute of Public Health, Srobarova 48, 10042 Prague, Czech Republic

**Keywords:** Eukaryotes, Genome, Gene structure, Longest intron, Gene function, Ribosome biogenesis, Spliceosome

## Abstract

Despite the fact that introns mean an energy and time burden for eukaryotic cells, they play an irreplaceable role in the diversification and regulation of protein production. As a common feature of eukaryotic genomes, it has been reported that in protein-coding genes, the longest intron is usually one of the first introns. The goal of our work was to find a possible difference in the biological function of genes that fulfill this common feature compared to genes that do not. Data on the lengths of all introns in genes were extracted from the genomes of six vertebrates (human, mouse, koala, chicken, zebrafish and fugu) and two other model organisms (nematode worm and arabidopsis). We showed that more than 40% of protein-coding genes have the relative position of the longest intron located in the second or third tertile of all introns. Genes divided according to the relative position of the longest intron were found to be significantly increased in different KEGG pathways. Genes with the longest intron in the first tertile predominate in a range of pathways for amino acid and lipid metabolism, various signaling, cell junctions or ABC transporters. Genes with the longest intron in the second or third tertile show increased representation in pathways associated with the formation and function of the spliceosome and ribosomes. In the two groups of genes defined in this way, we further demonstrated the difference in the length of the longest introns and the distribution of their absolute positions. We also pointed out other characteristics, namely the positive correlation between the length of the longest intron and the sum of the lengths of all other introns in the gene and the preservation of the exact same absolute and relative position of the longest intron between orthologous genes.

**Supplementary Information:**

The online version contains supplementary material available at 10.1186/s12864-024-10558-x.

## Introduction

Four distinct types are recognized among introns, generally defined as sequences within genes that are subsequently excised from the corresponding RNA transcripts [1, 2]. These are the so-called group I and group II self-splicing introns, transfer RNA introns and spliceosomal introns. Spliceosomal introns, which are the focus of this work, are excised from precursor mRNAs by spliceosome, a special ribonucleoprotein complex [3]. Introns of this type were found in the nuclear genomes of all representatives of the Eukarya domain investigated so far, but were not observed in any of the representatives of the other two domains – Bacteria and Archaea [4]. Part of a research devoted to introns is therefore focused mainly on clarifying questions about the origin and evolution of introns, another part is mostly concerned with unraveling the biological functions of introns in the genes of extant species.

As part of a long-standing scientific discussion, three main scenarios for the origin and evolution of introns have been formulated [4–8]. The introns-early theory assumes that introns were present in the common ancestor of all three domains of life—the last universal common ancestor (LUCA)—and were subsequently eliminated in the genomes of all representatives of bacteria and archaea. Similarly, some groups of eukaryotic organisms mainly lost introns, resulting in large differences in the number of introns embodied in the genomes of representatives living today.

In contrast, the introns-late theory places the origin of spliceosomal introns at a much later time, between the common ancestors of all modern eukaryotic organisms. The number of introns increased differently in diverse lineages mainly by the mechanism of reverse splicing and the insertion of transposable elements.

The third variant was brought by the theory called introns-first, which infers the presence of introns already at the very beginning of the creation of genes, even before the creation of DNA, in an environment in which all processes of transmission and implementation of hereditary information were mediated and controlled by RNA molecules, the so-called RNA world [9, 10].

The number and size of introns exhibit a great variability between the genomes of different present-day organisms. It was calculated that in the genomes of organisms with a lower intron density (approximately up to 3 introns per 1 kilobase pair, kbp) shorter introns (with an average length of about 75 bp) occur, without a significant correlation between density and length. On the contrary, in organisms with a higher density of introns, the positive correlation between density and length is already significant [8]. Among the so-called intron-poor organisms are a number of unicellular organisms, including, for example, the yeast Saccharomyces cerevisiae (with a density of about 0.05 introns per gene and an average length of 256 bp) [11]. The genomes of all vertebrates belong to the intron-rich group, of which the genomes of mammals show the highest density and length of introns (approximately 8 introns per gene with an average length of around 6 kbp) [6, 12]. In intron-rich organisms, we can find a great variability in the number and size of introns, even among individual genes.

As a general rule for most eukaryotic species studied, the first intron is the longest intron in a given gene. This rule is even more pronounced when the first intron is located in the 5' UTR region of the gene [13]. One of the reasons for the exceptional status of the first introns in a gene may be their increased association with affecting gene expression, which has already been suggested in analyses of various eukaryotic species [14, 15]. This phenomenon is often referred to as intron-mediated enhancement (IME) and it has even been suggested that introns with such a function could be used to activate gene expression in gene therapy [16].

Although data from whole-genome sequencing of a number of organisms are already available, their interpretation is still ongoing and incomplete. Also, some genomic characteristics have not yet been studied in detail in the literature. At the beginning of our work, we asked ourselves this question: What is the actual percentage of genes that fulfill the above-mentioned characteristic, i.e. that the longest intron in a gene is located among the first introns? In our initial work on a selection of protein-coding genes of the human genome, we showed that approximately 64% of the genes have the longest intron in the 1^st^ tertile of all introns in the gene, while 19% in the second and 17% in the third. Notable peaks were seen at the position in the middle of the gene and the last intron (5 and 6%, respectively) [17]. It was therefore clear that a non-negligible number of genes do not have the longest introns in the first positions. Consequently, we asked the second question: Do genes that have the longest intron in the 2^nd^ or 3^rd^ tertile of all introns show any specific functional characteristics compared to genes that have the longest intron in the 1^st^ tertile? Such a relationship was implied in our aforementioned work on a subset of human genes. An example of *DNA repair* genes, among which there is a significantly higher representation of genes with the longest intron in the 2^nd^ or 3^rd^ tertile, was demonstrated for human genes.

In this follow-up work, we set ourselves the task of finding out whether the relationships between the position of the longest intron and the biological function of genes have a more general validity among other eukaryotic organisms. Our analyses were therefore performed on genome-wide data from six representatives of vertebrates, as the group with the largest volume of biological knowledge, and two representatives of more distant model organisms as outgroups. We bring some new information about the longest introns in genes with a broader validity in this article.

## Materials and methods

### Primary data and species

Available data on all protein-coding genes of a given organism were downloaded from the Ensembl database (https://www.ensembl.org/index.html; Release 109) [18], from which the lengths of all introns were calculated according to the algorithm described below. The analyzed vertebrates included: human (*Homo sapiens*), mouse (*Mus musculus*), koala (*Phascolarctos cinereus*), chicken (*Gallus gallus*), zebrafish (*Danio rerio*) and fugu (*Takifugu rubripes*). Intron data from a nematode worm (*Caenorhabditis elegans*) and arabidopsis (*Arabidopsis thaliana*) were taken as outgroups. The MANE Select and Ensembl Canonical Flag features, which we set as selection criteria, has not yet been established for all genomes of organisms available in the given database, and therefore this characteristics was the main limitation for the selection of vertebrate organisms we could test (http://www.ensembl.org/info/genome/genebuild/transcript_quality_tags.html). Regarding the nematode worm data, the main gene isoforms have not yet been identified for this organism, therefore we analyzed the set of all gene transcripts included in the APPRIS system.

### Algorithm for calculating the lengths of introns

In the first step, we obtained the positions of the beginnings (hereinafter referred to as Exon Start) and ends (Exon End) of all exons (coding as well as non-coding exons in untranslated regions) in the desired transcripts of all protein-coding genes of the tested organism. For this, we used a query to the Ensembl database via the BioMart tool. In order to evaluate the most representative transcripts, we used MANE Select flags for Filters criteria in the human genome [19]. Considering that MANE Select is not available for other genomes, we sorted the transcripts in other organisms based on the selection of criteria Gene type—protein coding and at the same time Ensembl Canonical—only. Among the data (Attributes) we queried for each transcript there were: Gene stable ID, Gene stable ID version, Transcript Stable ID, Gene name, Strand, Exon rank in transcript, Exon region start (bp) and Exon region end (bp).

In the second step, we used an in-house shell script to calculate the lengths of individual introns from the obtained data, the code is available in the Supplementary Information as Introns2.5 (SH Source File) or Supplementary Information S1 (Microsoft Excel File). Genes were sorted not by their names (symbols) but by Gene or Transcript Stable IDs. We calculated intron lengths in bp for genes on the Forward strand according to the formula {[Exon(n + 1)Start – Exon(n)End] – 1}; n are positive integers starting from 1. For genes on the Reverse strand, this formula was modified to {[Exon(n)Start – Exon(n + 1)End] – 1}. The script then created a table with the lengths of all introns for each protein-coding gene and searched for the position of the longest intron in the gene. In particular, we used AWK language to perform the following steps: 1) Calculate the lengths of the introns; 2) Extract Gene names if they were available; 3) Indicate the longest intron; 4) Calculate the relative position of the longest intron (the ratio of the position of the longest intron to the total number of introns in the given gene). Then we used Bourne Again Shell (BASH) to create a matrix of Gene or Transcript Stable IDs versus the lengths of the introns in bp. The script can handle various delimiters and require an input file exported from Ensembl BioMart query with either Gene Stable ID or Transcript stable ID as the main unique identifier. The first seven columns in the file must be: (1) Gene stable ID, (2) Transcript stable ID, (3) Gene name, (4) Strand, (5) Exon rank in transcript, (6) Exon region start and (7) Exon region end. The primary data about length of introns obtained by this algorithm are stored for individual tested organisms in Supplementary Information Tables S2-9.

In case the gene contained several introns with the same longest value, the longest intron was chosen as the one that had the highest absolute position number, i.e., position furthest from the 5' end of the gene. In the genomes of tested vertebrates, this situation occurred only in less than 0.1% of genes, in nematode worm and arabidopsis in less than 1%. The list of genes for which this situation occurred is presented in Table S10.

### Gene set enrichment analysis (GSEA)

Based on our previous work with the human genome and the knowledge that the distribution of the positions of the longest introns showed three peaks—at the beginning, middle and end of the gene, we divided all protein-coding genes of the studied organism into those containing less than three introns and which contain three or more introns [17]. We further divided genes with three or more introns into three subgroups according to the relative position of the longest intron (defined above). Thus, the position of the longest intron in the 1^st^ tertile of all introns means that the relative ratio is in the interval (0;0.33]. Similarly, for the position of the longest intron in the 2^nd^ tertile, it is in the interval (0.33;0.66], and for the 3^rd^ tertile in (0.66;1]. In GSEA analyses, three subgroups of all protein-coding genes were compared from individual organisms; these input data for GSEA analyses can be obtained from Table S11.

We performed GSEA in parallel using two web platforms – g:Profiler (https://biit.cs.ut.ee/gprofiler/gost) [20] and ShinyGO (http://bioinformatics.sdstate.edu/go/) [21], taking into account the procedure recommended in the work of Reimand et al. [22]. With the g:Profiler program, we used the option to analyze multiple gene files simultaneously (*Run as multiquery* option), other possible options for setting the result parameters were left in the default settings. The default settings were also left in the ShinyGO program. We used the AmiGO 2 project (http://amigo.geneontology.org/amigo/landing) [23] for a better navigation in the hierarchical structure of GO terms (Gene Ontology; http://geneontology.org/) [24] in the resulting lists of terms and the tool for creating Venn diagrams Multiple List Comparator (https://molbiotools.com/listcompare.php) [25] for finding common terms between these lists.

### Random gene lists

For each set of the GSEA input data, we created equally large set of control data. The creation of these control data proceeded in such a way that all the protein-coding genes of the given organism were randomly divided into subgroups with the same number of genes as in the subgroups of the GSEA input data, where the genes were divided according to the relative position of the longest intron. The "shuf" command in the Linux operating system was used to randomize individual genes into these control sets, which are available in Table S12. A basic statistical comparison of the primary and control data sets showed the random selection of genes in the control data.

### Analysis of orthologous genes

The analysis of orthologous genes carried out by us aimed primarily to demonstrate whether a situation can occur between these genes where the same absolute or even relative position of the longest intron is preserved. Furthermore, we were interested in an approximate estimate of the frequency of this phenomenon and whether its localization within the gene, i.e. its relative position, can influence any change in the position of the longest intron. On the data on protein-coding genes obtained for the above-mentioned analyses, we monitored the change in the absolute and relative position of the longest intron in three subgroups of orthologous genes. From the human genes with the longest intron in the 1^st^, 2^nd^ and 3^rd^ tertiles, we randomly selected 20 genes from each group and searched for the corresponding orthologous genes in the tested vertebrates. Information available in the Ensembl database was used to assess the orthologous relationship. In cases of the presence of multiple orthologous genes in one species at the same time, only one of them was selected among these genes, according to the highest values ​​for Gene Order Conservation (GOC) score and the Whole Genome Alignment (WGA) coverage (https://www.ensembl.org/Help/View?id=135). Due to the random selection of genes, human genes that have no described orthologous genes in other vertebrate species were also selected for the analysis.

### Statistics and visualization

Freely available PAST software package (PAleontological STatistics, Natural History Museum, University of Oslo; version 4.11; https://www.nhm.uio.no/english/research/resources/past/) was used to evaluate the data using basic statistical methods for comparison of several univariate groups (e.g. the Kruskal–Wallis and Mann–Whitney pairwise tests). The usual threshold for statistical significance (*p* < 0.05) was accepted. Bonferroni adjustment or False Discovery Rate corrections were used during multiple testing depending on the availability of these calculations in the used programs. Hierarchical clustering with average linkage method and subsequent heatmap creation was performed in Morpheus web server (Broad Institute, Cambridge, USA; https://software.broadinstitute.org/morpheus). Tree-map visualizations of lists of GO terms were created with Revigo software (http://revigo.irb.hr/) [26]. KEGG diagrams (Kyoto Encyclopedia of Genes and Genomes; https://www.genome.jp/kegg/) [27] with genes highlighted were created with the help of Pathview (https://pathview.uncc.edu/) [28].

## Results

### More than 40% of protein-coding genes have the longest intron located in the 2^nd^ or 3^rd^ tertiles

Among the six analyzed vertebrate genomes, there is up to a two-fold difference in the number of annotated protein-coding genes, the lowest number in chicken (16,711) and the highest in zebrafish (30,153). However, the intron density is comparable, i.e. 6 or 7 introns per one gene (median). The representation of intronless genes ranges from 3% in chicken to 7% in mouse and koala. The percentage of genes with three or more introns ranges between 73 and 80% in these genomes. Regarding the position of the longest intron, the longest intron in the 1^st^ tertile had 44% (zebrafish) to 58% (human) of the genes with three or more introns. Compared to the tested vertebrates, the intron density value of worm is 5 and the percentage of genes with the longest intron in the 1st tertile is only 39%. In arabidopsis, the intron density is even lower (3), and genes with three or more introns make up only 51% of all protein-coding genes, of which 49% have the longest intron lying in the 1^st^ tertile. Table 1 provides an overview of the analyzed genomes.

**Table 1:** Overview of analyzed genomes

Number (Percentage)	Human	Mouse	Koala	Chicken	Zebrafish	Fugu	Worm	Arabidopsis
Protein-coding genes	19,060 (100)	21,959 (100)	19,832 (100)	16,711 (100)	30,153 (100)	21,383 (100)	27,811 tr. (100)	27,628 (100)
Intron density (average/median)	10/7	9/6	8/6	10/7	9/6	9/6	6/5	4/3
Intronless genes	1024 (5)	1478 (7)	1466 (7)	563 (3)	1406 (5)	989 (5)	763 (3)	5779 (21)
Genes with 1 intron	1352 (7)	2144 (10)	2161 (11)	1530 (9)	2567 (9)	1942 (9)	2380 (9)	4556 (16)
Genes with 2 introns	1408 (7)	1877 (9)	1805 (9)	1317 (8)	2748 (9)	1900 (9)	3339 (12)	3223 (12)
Genes with 3 and more introns	15,276 (80)	16,460 (75)	14,400 (73)	13,301 (80)	23,432 (78)	16,552 (77)	21,329 (77)	14,070 (51)
**Relative position of the longest intron**^a^								
1^st^ tertile	8873 (58)	9368 (57)	7965 (55)	7284 (55)	10,384 (44)	8929 (54)	8217 (39)	6860 (49)
2^nd^ tertile	3201 (21)	3651 (22)	3135 (22)	2875 (22)	5545 (24)	3482 (21)	6132 (29)	3908 (28)
3^rd^ tertile	3202 (21)	3441 (21)	3300 (23)	3142 (24)	7503 (32)	4141 (25)	6980 (33)	3302 (23)

*tr *Transcripts

^a^among genes with 3 and more introns

### The longest introns located in the 1^st^ tertile of all introns in the gene are significantly longer than the longest introns in the 2^nd^ or 3^rd^ tertile

**Fig. 1:**
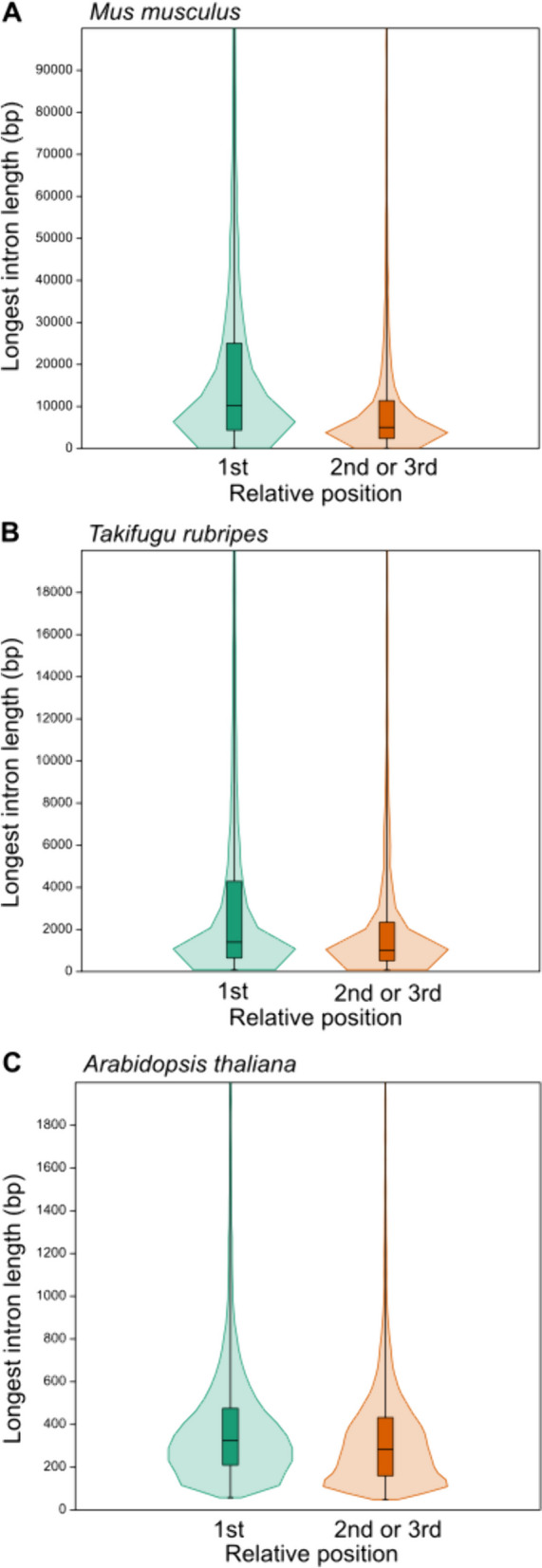
Comparison of longest intron lengths for genes with the longest intron position in the 1^st^ versus 2^nd^ or 3.^rd^ tertile for mouse (**A**), fugu (**B**) and arabidopsis (**C**). The median is shown with a horizontal line inside the boxes. In all species, there is a significant statistical difference between the two tested groups (*p* < 0.0001, Mann–Whitney test)

By dividing the protein-coding genes into a group with the longest intron located in the 1^st^ tertile and a group with the longest intron in the 2^nd^ or 3^rd^ tertile, two approximately equally numerous groups will be created in all tested species. For the two groups of genes defined in this way, we compared the lengths of the longest introns. In all analyzed genomes of vertebrates and two other model organisms, the group with the longest intron in the 1^st^ tertile showed significantly longer introns. The results of this analysis are shown for selected species in Fig. 1, and numerical values for all species are summarized in Table S13.

### The tested groups of genes according to the relative position of the longest intron show a different distribution of absolute positions

**Fig. 2:**
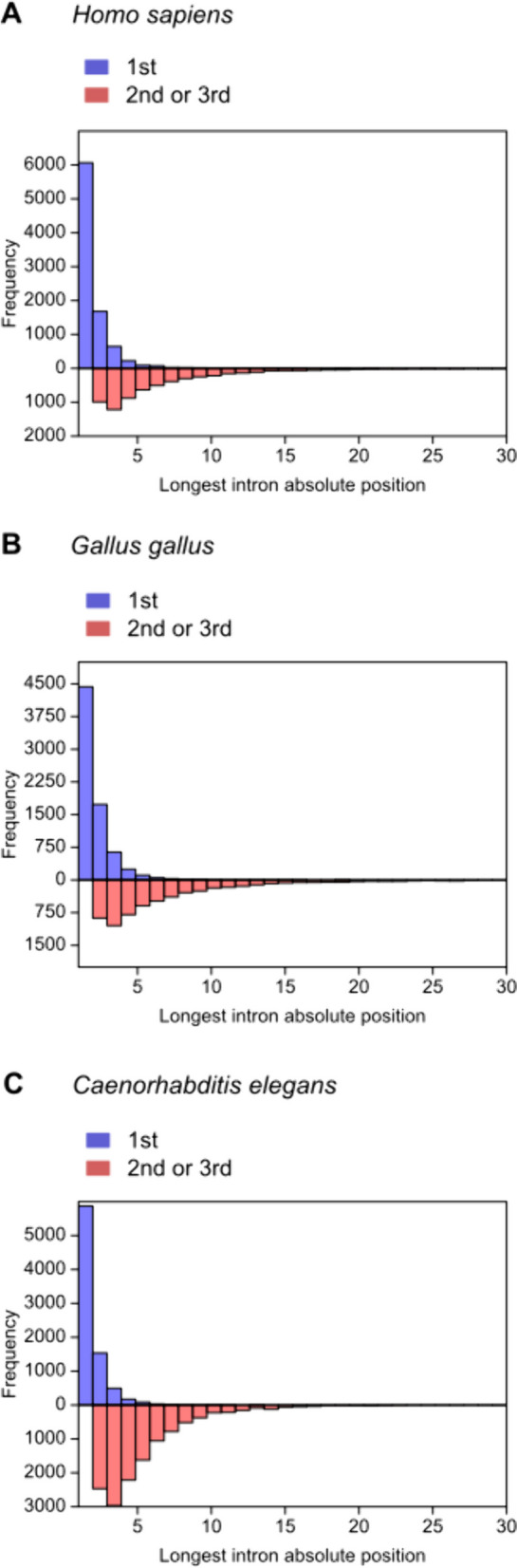
Distribution of the longest intron absolute positions in the two compared groups of genes defined by the relative positions of these introns (1^st^ versus 2^nd^ or 3^rd^ tertile)

Groups of genes divided by the relative position of the longest intron in the 1^st^ versus 2^nd^ or 3^rd^ tertile differ in the representation profile of the absolute positions of these introns. For all tested species, in the 1^st^ tertile group, the percentage of the intron with the absolute position No. 1 ranged between 61 and 77%, intron No. 2 between 15 and 24%, and intron No. 3 between 4 and 9%. In the 2^nd^ or 3^rd^ tertile group, no introns with the absolute position No. 1 were present and the percentage representation of the longest introns with the absolute position No. 2, 3, 4, 5, 6 and 7 was in the range of 13–21%, 17–24%, 13–17%, 10–12%, 7–9% and 6–7%, respectively. Histograms showing the described distributions are shown for selected species in Fig. 2, the calculated values are disclosed for all species in Table S14.

### All tested species showed a positive correlation between the lengths of the longest introns and lengths of all other introns in genes

**Fig. 3:**
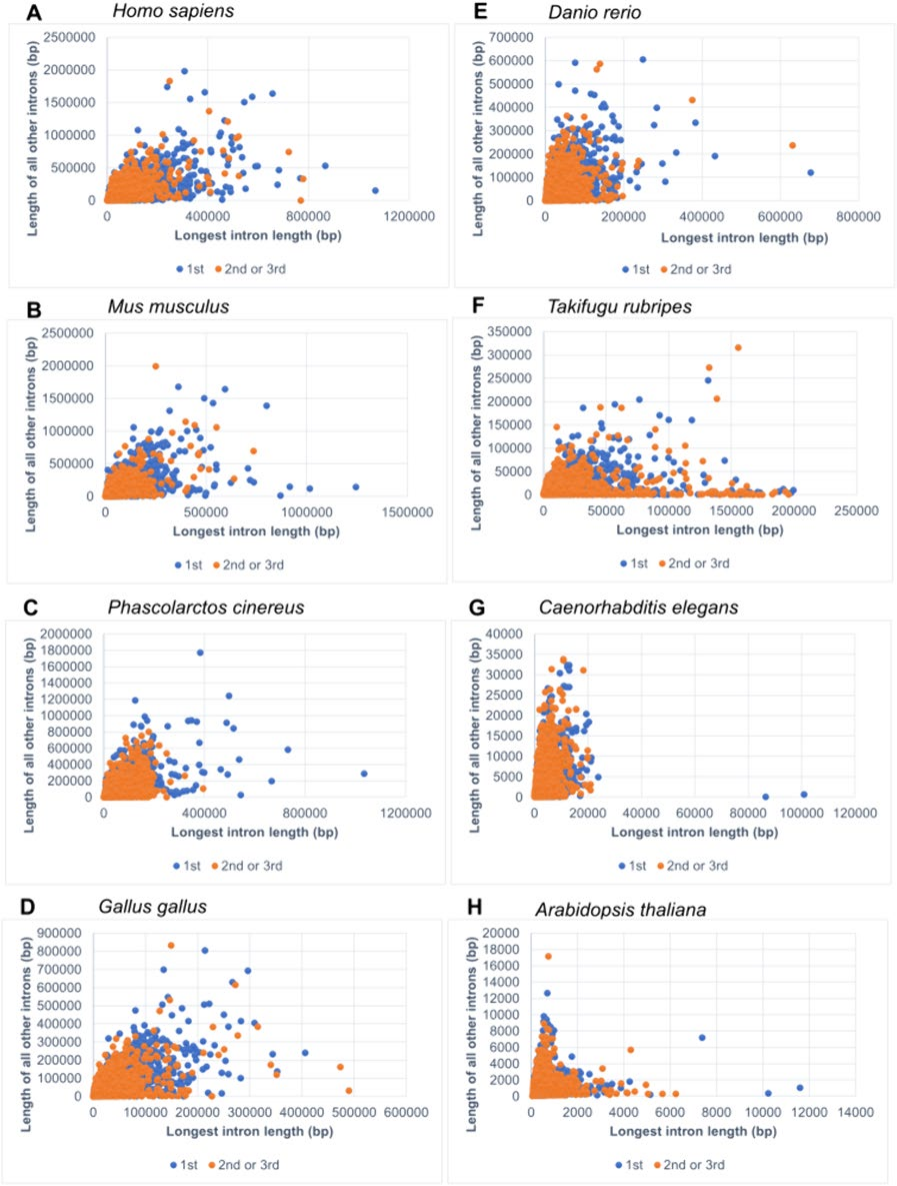
Scatter plots showing the correlation between the length of the longest intron and the sum of the lengths of all other introns in the gene. Protein-coding genes in human (**A**), mouse (**B**), koala (**C**), chicken (**D**), zebrafish (**E**), fugu (**F**), nematode worm (**G**) and arabidopsis (**H**) were tested, with overall Spearman rs coefficients 0.8, 0.77, 0.79, 0.79, 0.77, 0.72, 0.76 and 0.33, respectively; the corresponding *P* values were less than 0.0001 in all cases

A correlation analysis between the variables – the length of the longest intron and sum of the lengths of all other introns in the gene – was performed. Firstly, all protein-coding genes in genomes were evaluated together. Correlation coefficients (Spearmen's rs) ranged from 0.72 to 0.8 (*P* values less than 0.0001) for all vertebrates and nematode worm. These values can be interpreted as a strong association. In arabidopsis, the same coefficient had a value of 0.33 (*P* < 0.0001). This result expresses only a weak relationship. Secondly, the two subgroups – 1^st^ versus non-1^st^ tertile – were evaluated separately. A similar correlation patterns as well as coefficient values were detected for both subgroups as for the sets with all genes. Specific differences can be observed in the individual scatter plots, which are related to different lengths of introns in evolutionarily more distant groups (Fig. 3). However, a positive correlation between the two variables can be considered a general trend, although it was stronger in animals than in the one member of plants tested.

### Genes divided according to the relative position of the longest intron show increased representation in different KEGG pathways

GSEA analyses were performed separately for genes with one and two introns and for genes with three or more introns. Between the significant results for the individual tested organisms, their intersections were further sought with the intention of defining the most general trend. The koala was excluded from these analyses because this species still lacks sufficient data in the Gene Ontology and KEGG pathways databases.

For genes with one or two introns, only one significantly increased KEGG pathway—*Neuroactive ligand-receptor interaction*—was found common to all 5 tested vertebrates. The *Cytokine-cytokine receptor interaction* pathway was significant for 3 vertebrates (human, mouse and zebrafish).

**Fig. 4:**
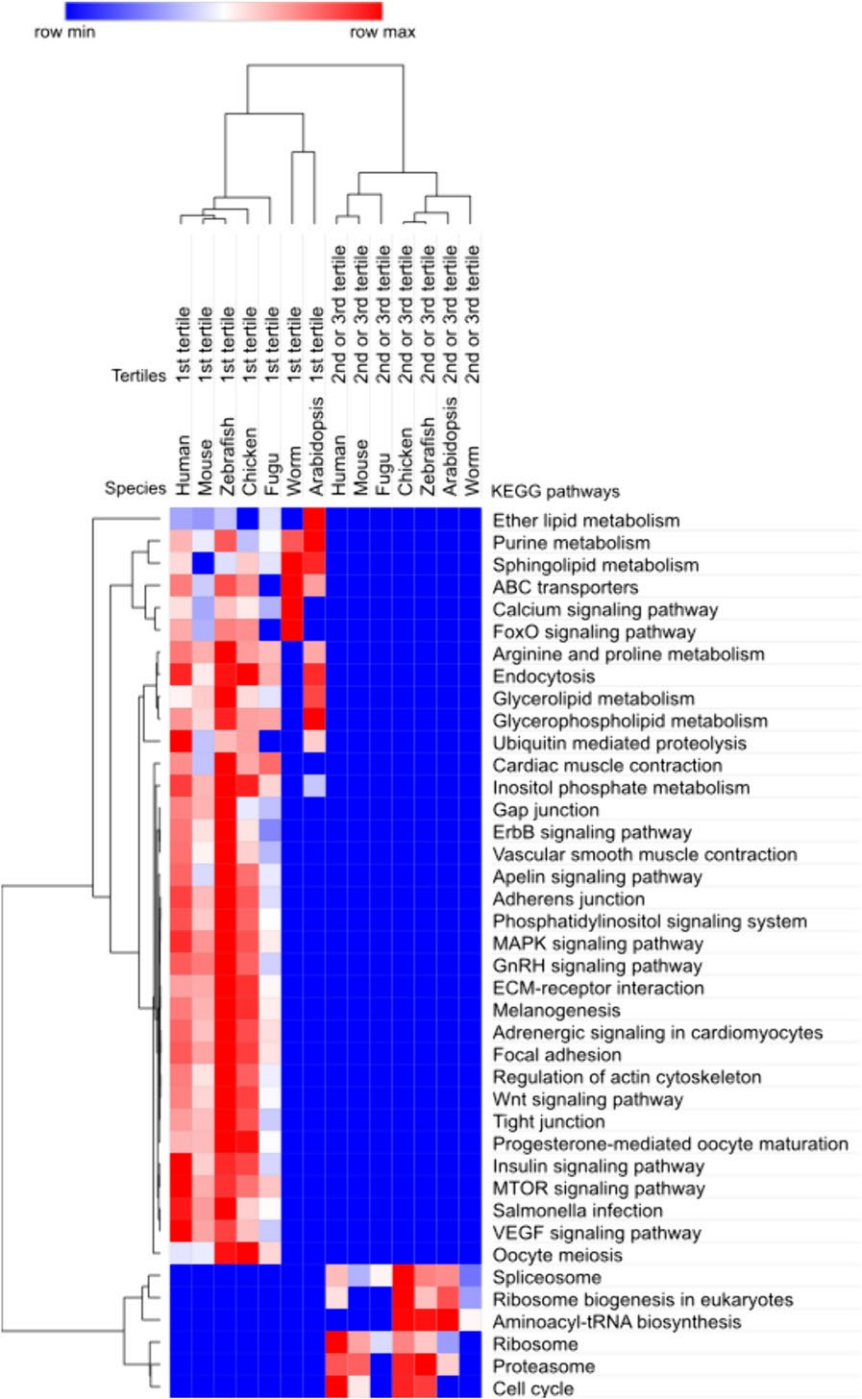
KEGG pathways significantly overrepresented in genes with the longest intron in 1^st^ versus 2^nd^ or 3^rd^ tertile of introns

For genes with three or more introns, the same two subgroups as in the previous analyses were tested – genes with the longest intron in the 1^st^ versus 2^nd^ or 3^rd^ tertile. Intersections between the results in individual species showed that the 1^st^ tertile group is most generally characterized by the following pathways: *ABC transporters, Arginine and proline metabolism, Calcium signaling pathway, Endocytosis, Glycerolipid metabolism, Glycerophospholipid metabolism, Inositol phosphate metabolism, Purine metabolism,* and *Sphingolipid metabolism*. All these pathways were found in the intersection of at least six tested organisms, at least one of which was a representative from the outgroups. Significantly, the pathways *Spliceosome*, *Ribosome*, *Proteasome*, and *Ribosome biogenesis in eukaryotes* are characteristic for the genes with the longest intron in the 2^nd^ or 3^rd^ tertile. For these pathways, we found a match in all seven tested organisms for *Spliceosome*, for the others there was a match in at least five organisms, at least one of which was from outgroup species. The results of GSEA analyses and their visualization are presented in Table S15 and Fig. 4.

### Genes with the longest intron in the 2^nd^ or 3^rd^ tertile are found among all components of the spliceosome

**Fig. 5:**
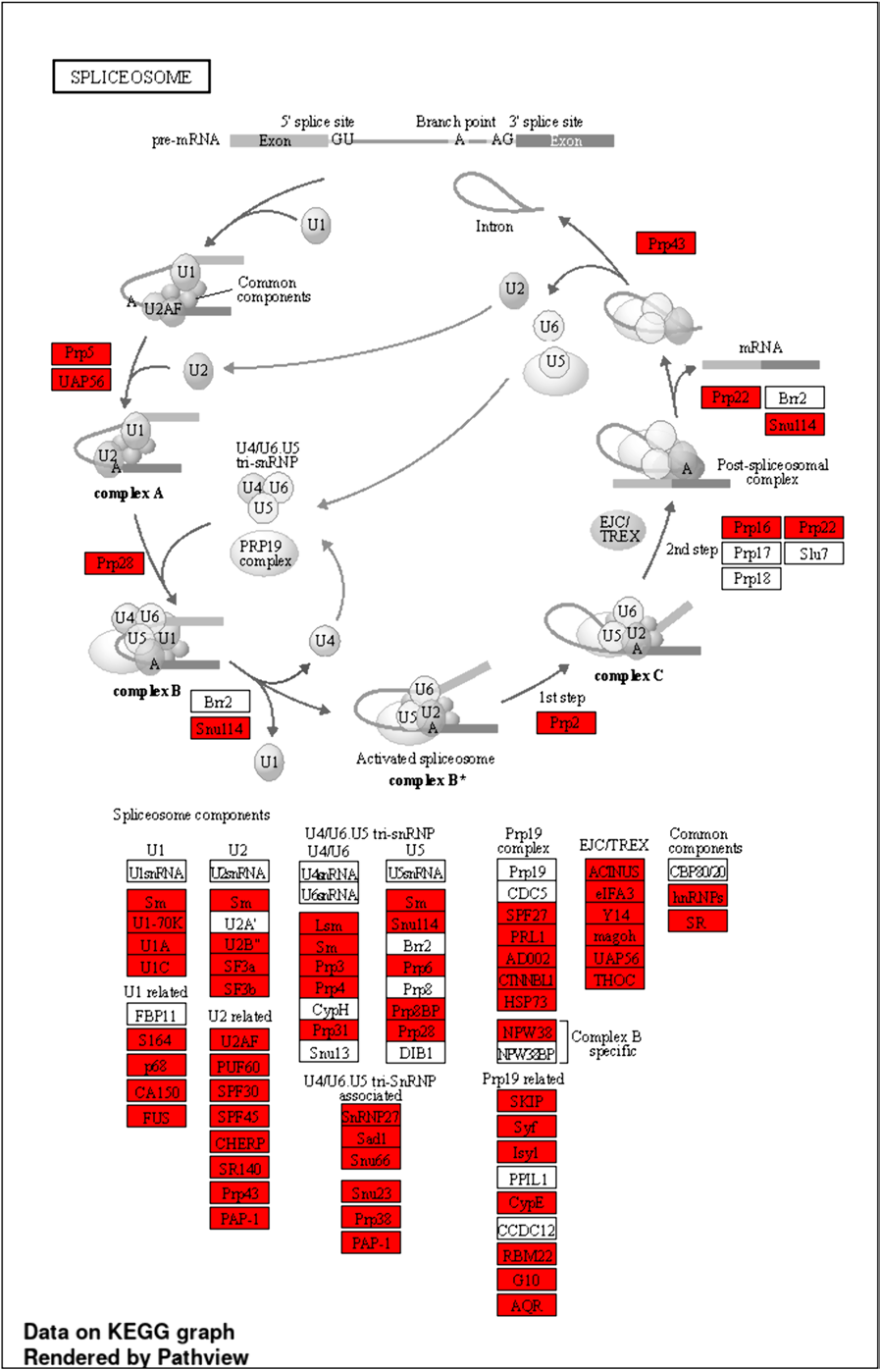
The Spliceosome pathway in the genome of the model organism zebrafish. The genes, which have the longest intron located in the 2^nd^ or 3^rd^ tertile of all introns are highlighted in red

For the *Spliceosome* pathway, we took a closer look at which specific genes belong to the genes with the longest intron in the 2^nd^ or 3^rd^ tertile and also in which of the tested species this characteristic is preserved. We can conclude that all Spliceosome components contain proteins whose genes fall into the mentioned characteristic. Seven out of 70 monitored components retained this characteristic in all 7 tested organisms and 15 components in 6 organisms. Among the most conserved components of the spliceosome are: Sm, Lsm, U1-70K, U1C, p68, U2B, SF3a, SF3b, Prp8BP, Sad1, Prp38, PRL1, Syf, G10, AQR, Y14, THOC, hnRNPs, and SR. Figure 5 provides a visualization of this analysis for zebrafish.

### Control data did not show the same repeatability of significant results across organisms as did the genomic data

Following the same procedure as for the primary data, GSEA analyses were also performed with the control gene sets. Due to the nature and amount of tested data, some GO terms, but not pathways, were found to be statistically significant for individual organisms, even for the control sets. However, these were always terms other than those that were significant in the primary genomic data, and above all none of these GO terms was replicated in any other of the tested organisms. The recurrence of the same GO terms and pathways among different species in the primary data is a strong argument for the biological significance of the results presented here.

### A phenomenon where the same absolute and relative position of the longest intron is preserved among orthologous genes is rare

From 60 randomly selected human genes and their respective orthologous genes in 5 other vertebrate representatives, we demonstrated the preservation of the same absolute position of the longest intron in 3 genes (*ACAP2, LMCD1* and *NPAS4*). In all these cases, it was the first intron in the gene. From the point of view of the relative position of the longest intron, we observed the preservation of exactly the same value among all found orthologous genes only in 1 gene (*NPAS4*). We observed preservation of the relative position within the same tertile group for 8 genes from the 1^st^ tertile group, 2 genes from the 2^nd^ tertile group and no genes from the 3rd tertile group. All genes included in this analysis and monitored values ​​are recorded in Table S16, visualization of the results is provided in Figure S1.

## Discussion

Although longer introns represent an energy and time burden for the genetic apparatus of the cell trying to efficiently transfer information from its storage to concrete implementation, they perform important and apparently irreplaceable tasks in this process. The results of whole-genome sequencing of many organisms, which have recently increased significantly and become freely available, have enabled our deeper understanding of genome organization. Due to the complexity of this issue, however, there are still many unanswered questions. Our work shows the relationship between genes with a certain biological function and the location of the longest intron within the exon–intron structure of the gene. According to the data presented, the differences are evident even when the genes are roughly divided into genes with the longest intron located in the 1^st^ tertile of all introns and genes with the longest intron in the 2^nd^ or 3^rd^ tertile. In the first of these two groups, genes from a wide spectrum of biological functions, primarily associated with the development of more complex multicellular organisms, predominate. In the second group, a higher representation of genes associated with the biogenesis and function of the spliceosome and ribosomes can be demonstrated.

The presence of longer introns closer to the 5' end of eukaryotic genes is a well-known phenomenon [13], which is explained by a greater occurrence of regulatory elements and thus a greater restriction of selectivity in these introns. There is evidence that longer introns in general contain higher densities of conserved sites [29] and various regulatory elements [15, 30]. However, introns of thousands to tens of thousands of base pairs already represent a significant burden for the process of transcription and splicing, and therefore other functional advantages of this system are sought. Prolonged transcription time in the case of long introns has been proposed as one of the mechanisms influencing the resulting gene expression [31].

Splicing of long introns must overcome the problem of considerable physical distance between the sequences involved. In long introns in invertebrate organisms, combined donor–acceptor splicing sites (called RP-sites) were detected to an increased extent, and a process of gradual removal of smaller sections named recursive splicing was proposed [32]. However, in long introns of vertebrates, these RP-sites were not observed to an increased extent, and therefore modified solutions were suggested. Shepard et al. [33] recognized an increased amount of SINE and LINE repetitive sequences in long introns of vertebrates and proposed the formation of multiple hairpins with large loops. These hairpins can form compact spatial structures facilitating splicing. Kelly et al. [34] further studied other possibilities of recursive splicing in vertebrates and found usage of RP-sites with alternative sequences.

It turns out that different exon–intron architecture is used by different groups of genes according to the biological context. If we compare housekeeping genes with tissue-specific genes, the length of housekeeping genes is significantly smaller [35]. Similarly, genes whose products are used in a rapid biological response have lower intron density than genes whose products are applied after a certain time delay [36, 37]. Our results correspond with those reported in the work of Schonfeld and colleagues [38]. Using a computer model, they showed that the introns of essential genes show such specific characteristics that essential genes can be defined and distinguished from non-essential genes. Their work focused primarily on the first introns, where they demonstrated that essential genes have significantly shorter introns than non-essential genes.

The RNA world theory deals with the explanation of the evolutionary development of the exon–intron organization of current genes [39]. This theory considers the existence of RNA and its essential function for the transfer of genetic information and its implementation among the first cells on our planet even before the existence of DNA and the function of proteins as catalysts. Among other things, this theory considers the irreplaceable role of introns, which also existed already at this initial stage [40, 41]. The later incorporation of additional introns in the form of transposable elements with no initial function for gene expression has created a very heterogeneous intron system that is complex to understand and reveal from our current situation [5, 42]. As a consequence, a certain gene architecture most likely promotes or suppresses gene evolution [43].

The main limitation of our work is a certain degree of simplification given by following only the main isoforms of protein-coding genes and neglecting the influence of alternative splicing. In addition to the targeted simplification of the whole situation, our approach was also guided by the so far limited amount of knowledge about the biological function of other than the main isoforms. Therefore, expanding the data to include other gene isoforms and more accurately scaling the length of the longest introns could be the next direction for follow-up research. Also, the analysis of the conservation of the lengths of the longest introns between orthologous genes, which was done in this work only on a limited sample of genes, could, with the extension to whole-genome data, bring other new and interesting findings in the future.

### Supplementary Information

Supplementary Material 1. Figure S1: Comparison of the absolute and relative positions of the longest introns among orthologous genes of 6 vertebrates. Twenty randomly selected human genes and their corresponding orthologs were monitored in each of the groups with the longest human intron in the 1^st^ tertile (A), 2^nd^ tertile (B) and 3^rd^ tertile (C).

## Data Availability

The datasets—code as well as all Supplementary Information—generated during and/or analysed during the current study are available in the Zenodo repository, 10.5281/zenodo.12577986.
