# Supplementary figures and images for: Genes divided according to the relative position of the longest intron show increased representation in different KEGG pathways
Source: BMC Genomics. 2024 Jun 28;25:649. doi: 10.1186/s12864-024-10558-x (PMC11214234; doi:10.1186/s12864-024-10558-x)

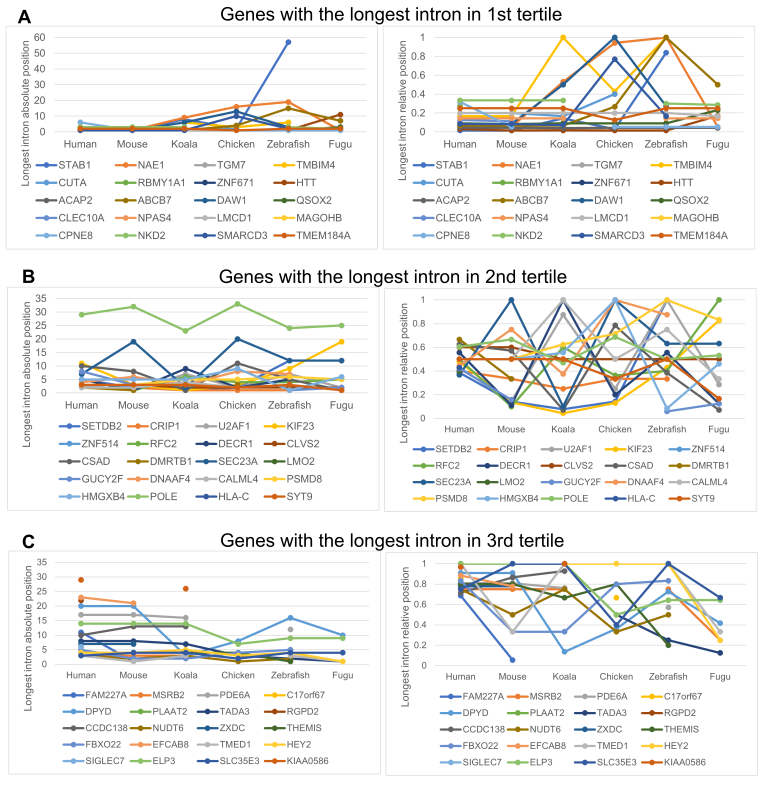

Supplement: Supplementary file 1 — Supplementary Material 1. Figure S1: Comparison of the absolute and relative positions of the longest introns among orthologous genes of 6 vertebrates. Twenty randomly selected human genes and their corresponding orthologs were monitored in each of the groups with the longest human intron in the 1st tertile (A), 2nd tertile (B) and 3rd tertile (C). [file 12864_2024_10558_MOESM1_ESM.zip › Supplementary Information Dvorak/Figure S1_v1_Longest intron positions among ortholog genes.png]
